# Finding consistent disease subnetworks across microarray datasets

**DOI:** 10.1186/1471-2105-12-S13-S15

**Published:** 2011-11-30

**Authors:** Donny Soh, Difeng Dong, Yike Guo, Limsoon Wong

**Affiliations:** 1 National University of Singapore, 13 Computing Drive, Singapore 117417; 2 Imperial College London, 180 Queens Gate, London SW7 2BZ, UK; 3 Institute for Infocomm Research, 1 Fusionopolis Way, 21-01 Connexis (South Tower), Singapore 138632

## Abstract

**Background:**

While contemporary methods of microarray analysis are excellent tools for studying individual microarray datasets, they have a tendency to produce different results from different datasets of the same disease. We aim to solve this reproducibility problem by introducing a technique (SNet). SNet provides both quantitative and descriptive analysis of microarray datasets by identifying specific connected portions of pathways that are significant. We term such portions within pathways as “subnetworks”.

**Results:**

We tested SNet on independent datasets of several diseases, including childhood ALL, DMD and lung cancer. For each of these diseases, we obtained two independent microarray datasets produced by distinct labs on distinct platforms. In each case, our technique consistently produced almost the same list of significant nontrivial subnetworks from two independent sets of microarray data. The gene-level agreement of these significant subnetworks was between 51.18% to 93.01%. In contrast, when the same pairs of microarray datasets were analysed using GSEA, t-test and SAM, this percentage fell between 2.38% to 28.90% for GSEA, 49.60% tp 73.01% for t-test, and 49.96% to 81.25% for SAM. Furthermore, the genes selected using these existing methods did not form subnetworks of substantial size. Thus it is more probable that the subnetworks selected by our technique can provide the researcher with more descriptive information on the portions of the pathway actually affected by the disease.

**Conclusions:**

These results clearly demonstrate that our technique generates significant subnetworks and genes that are more consistent and reproducible across datasets compared to the other popular methods available (GSEA, t-test and SAM). The large size of subnetworks which we generate indicates that they are generally more biologically significant (less likely to be spurious). In addition, we have chosen two sample subnetworks and validated them with references from biological literature. This shows that our algorithm is capable of generating descriptive biologically conclusions.

## Background

There is a wealth of techniques for identifying significant differential gene expression. These techniques can be categorized into three approaches; viz., individual genes, gene pathways and gene classes approaches.

• Individual genes

These techniques search for individual genes that are differentially expressed. For example, the fold change, t-test and Significance Analysis of Microarrays (SAM) [1]. The output of such algorithms is a list of genes that are deemed differentially expressed.

• Gene pathway deduction

Methods of this genre attempt to infer biological information from data without using pre-existing biological information. Bayesian learning [2] and Boolean network learning [3] are representatives of this approach. The researcher will obtain a set of gene networks connected and inferred solely from the gene expression data.

• Gene classes

These techniques test how gene classes behave as a whole. These techniques either pre-process or post-process their information with existing biological background knowledge to guide their analysis of the microarray data. Examples include over-representation analysis (ORA) [4], Functional Class Scoring (FCS) [5], GSEA [6], NEA [7] and ErmineJ [8]. Results from such methods are normally a list of pathways or gene groups that are differentially expressed according to the algorithms.

The commonly acknowledged challenge of these techniques is obtaining reproducible results. For instance, in differentially expressed gene discovery, there should be a substantial overlap in the gene lists from different datasets of the same disease. This is inferred from the premise that similar underlying conditions cause the onset of certain diseases. However it has been shown that there is little concurrence among such gene lists [9-11].

For example, [11] demonstrated this inconsistency using SAM. For a pair of datasets involving prostate cancer [12,13], he calculated the percentage overlap of differentially expressed genes between them. The top 10 genes had a percentage overlap of 30% while the top 100 genes had a percentage overlap of 15%. The same calculations were repeated for lung cancer [14,15] and DMD [16,17] datasets, yielding similar low percentages.

In addition, the functional gene lists, pathways or classes determined by such methods do not provide sufficient descriptive information about the interplay and relationship of genes [18]. Hence the generated hypotheses are usually too general, rendering them ineffective in guiding further research and treatment [19].

In this article we present our technique, SNet, to identify subnetworks which are expressed significantly within a phenotype of a microarray experiment. Furthermore, we demonstrate the consistency—and thus reproducibility—of the identified subnetworks by achieving a high overlap (51.18% to 93.01%) between significantly differentially expressed genes (found within the identified subnetworks) of different microarray experiments of the same disease. Finally, we show that the significant genes found by t-test/GSEA formed much smaller subnetworks (<5 genes) than ours. These experiments demonstrate the consistency, reproducibility, descriptive power, interpretability and significance of subnetworks obtained using our technique.

## Approach

We hypothesize that specific biological processes within pathways are relevant to specific diseases. Thus our approach concentrates on identifying these biological processes that we termed “subnetworks”. These subnetworks should be largely the same across independent datasets of the same disease. Because the probability of such a subnetwork of highly expressed genes randomly occurring is sufficiently low, we are able to conclude that these subnetworks have a strong biological relevance with respect to the disease. Furthermore, such a subnetwork provides intricate information on the interplay and relationship between the genes, which will be advantageous in guiding subsequent research. This technique also removes sporadic genes that appear solitary within a biological pathway (because of their higher possibility of being a false positive).

We define the term “subnetwork” as “a set of genes and relationships where all genes in the subnetwork are reachable by all other genes in the (undirected) subnetwork. Reachability between genes is established by the existence of an undirected path between the genes of the subnetwork.” This is analogous to the definition of “connected components” in graph theory [20].

**Figure 1:**
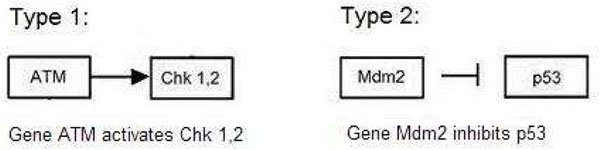
**Example of the two gene-gene relationships.** Example of the two gene-gene relationships. Left: an activating relationship between ATM and CHK1. Right: an inhibiting relationship between MDM2 and p53.

Only two types of gene-gene relationships are considered: inhibition and activation. This information regarding the relationship of two genes within a pathway is already inherent within the database and our goal is to find out if the microarray data complies with these relationships. In the example in Figure 1, we see the genes ATM, CHK1, CHK2 and MDM2 with the relationships: ATM activating CHK1, CHK2 and MDM2 inhibiting p53. Thus we define the term “relationship” between a pair of genes X and Y as a situation where either X “activates” Y or X “inhibits” Y.

Because of the fine granularity of analysis, the pathway repository must allow us to easily segregate the original microarray data into its relevant pathways, gene relationships and subnetworks. Due to the large amount of data, the pathway repository must also facilitate the development of automated analysis workflows. The repository therefore is required to have the following characteristics:

• Gene annotations have to be consistent with that in microarray experiments.

• Individual gene relationships within pathways have to be provided.

• The database must have a programmatic interface to access the data.

This set of criteria eliminates contemporary pathway sources such as Ingenuity [21], BioPax [22], and GenMapp [23], and we are left with KEGG. However, KEGG has a number of limitations. Firstly, its collection of pathways is not sufficiently comprehensive [24]. For example, our analysis [25] shows that 78.8% of pathways in Ingenuity and 64.4% of pathways in Wikipathways are not contained in KEGG. Secondly, KEGG still uses an old-fashioned SOAP/XML interface. So we developed PathwayAPI [25] which offered the combined pathway information of KEGG, Ingenuity, and Wikipathways along with a modern JSON-based application programming interface.

Our technique (to be described later) was applied on the disease types listed below with two different datasets analyzed independently for each disease type. The selection of the two datasets for each disease is because they were used to compare gene selection methods in earlier papers [11]. In addition, the two datasets for each disease type are from different platforms, thus providing a more stringent test as they make it harder for the gene selection algorithms to consistently select the same genes independently from the two datasets.

• Leukaemia: Comparison between leukaemia subtypes ALL and AML. Golub et al. [26] uses the Affymetrix HU6800 GeneChip with 47 ALL and 25 AML patients. Armstrong et al. [27] uses the Affymetrix HG-U95Av2 GeneChip with 24 ALL patients and 24 AML patients.

• Childhood Acute Lymphoblastic Leukaemia (ALL) Subtype: Comparison between two subtypes of childhood ALL leukaemia, namely E2A-PBX1 and BCR-ABL. Ross et al. [28]) uses the Affymetrix HG-U95Av2 GeneChip with 15 BCR-ABL patients and 27 E2A-PBX1 patients. Yeoh et al. [29] uses the U133A GeneChip with 15 BCR-ABL patients and 18 E2A-PBX1 patients.

• Duchenne Muscular Dystrophy (DMD): Comparison between patients suffering from DMD and normal patients. Haslett et al. [17] uses the Affymetrix HG-U95Av2 GeneChip while Pescator et al. [16] uses HG-U133A GeneChip. Haslett et al.’s dataset contains 24 samples from 12 DMD patients and 12 unaffected controls and Pescatori et al.’s consists of 36 samples from 22 DMD patients and 14 controls.

• Lung Cancer (Squamous): Comparison between patients suffering from squamous cell lung carcinomas and normal patients. For lung cancer, the cDNA microarray data consists of 13 samples with squamous cell lung carcinomas and five normal lung specimens [14], while the data by Affymetrix human U95A oligonucleotide arrays consist of 21 squamous cell lung carcinomas and 17 normal lung specimens [15].

## Results and discussion

### Significant subnetworks overlap

For each disease, two lists of significant subnetworks were identified by applying our technique (SNet) independently on the two different datasets for the disease. We next calculate the percentage overlap between the two lists of significant subnetworks.

This result is compared with another algorithm (GSEA) that extracts significant gene lists from microarray data. The individual pathways from the database (PathwayAPI [25], 386 pathways in total) and their associated genes are used as input gene sets for GSEA. Hence running GSEA with this database of pathways gives us a selected set of pathways deemed as significant by GSEA. GSEA is applied to both datasets of the same disease. For each dataset, we obtain a list of pathways significantly expressed and remove the pathways whose FDR q-value falls below 0.25. Finally, we calculate the percentage intersection between the remaining pathways within these two lists.

Results indicate that our technique consistently gives a higher percentage overlap for different datasets of the same disease than GSEA. Here, our technique obtained a high overlap percentage for these datasets (47.63% to 90.90%). As an example from Table 1, the percentage overlap of pathways in determining the ALL Subtype (second row in the table) in SNet is 47.63% while that for GSEA is 23.1%. The full results can be observed in Table 1. Table 2 shows the number of overlapping significant pathways for each disease type.

**Table 1:** Percentage overlap significant subnetworks between the datasets

Disease	Dataset 1	Dataset 2	SNet	GSEA
Leukaemia	Golub	Armstrong	83.33%	0%
ALL Subtype	Ross	Yeoh	47.63%	23.1%
DMD	Haslett	Pescatori	58.33%	55.6%
Lung	Bhattacharjee	Garber	90.90%	0%

Table showing the percentage overlap significant subnetworks between the datasets. Each row refers to a separate disease (as indicated in the first column). Each disease is tested against two datasets depicted in the second and third column. The overlap percentages refer to the pathway overlaps obtained from running SNet (column 4) and GSEA (column 5).

**Table 2:** Number of overlap significant subnetworks between the datasets

Disease	Dataset 1	Dataset 2	SNet	GSEA
Leukaemia	Golub	Armstrong	20	0
ALL subtype	Ross	Yeoh	10	6
DMD	Haslett	Pescatori	7	10
Lung	Bhattacharjee	Garber	9	0

Table showing the number of significant overlapping subnetworks between the significant pathways. Each row refers to a separate disease (as indicated in the first column). Each disease is tested against two datasets depicted in the second and third column. The overlapping figures refer to the pathway overlaps obtained from running SNet (column 4) and GSEA (column 5).

### Significant genes overlap

To demonstrate that the genes within the subnetworks are consistent across the datasets of the same disease, we obtained independently a list of significant genes from each dataset using SNet, GSEA, SAM and the t-test. After which we would calculate the percentage overlap between the same disease of each dataset. Results demonstrate that our SNet algorithm has a much higher overlap percentage as compared to the other techniques surveyed.

For SNet, we select the significant genes from each dataset by simply taking the genes from the subnetworks generated from each dataset. (As there are two independent datasets for each disease type, we generate two gene lists for each disease type. We denote the number of genes in the smaller list as γ). For GSEA, we obtain the list of significant genes by first selecting the top γ number of leading edge set of genes from the well expressed pathways for each dataset. The lists of significant genes for SAM and t-test are obtained by selecting all the genes with a p-value less than 0.05, as well as by selecting the top γ significant genes. The results shown in Table 3, Table 4 and Table 5 show that the gene overlap obtained from GSEA, t-test and SAM are consistently and significantly lower (2.38% to 28.90% for GSEA, 49.60% to 73.01% for t-test, 49.96% to 81.25% for SAM) compared to that of SNet (51.18% to 93.01%).

**Table 3:** Number and percentage of overlap genes

		SNet	GSEA
Leukaemia	Num Genes Genes overlap	*γ* =84 91.30%	84 2.38%

ALL subtype	Num Genes Genes overlap	*γ* =75 93.01%	75 4.0%

DMD	Num Genes Genes overlap	*γ* =45 69.23%	45 28.9%

Lung	Num Genes Genes overlap	*γ* =65 51.18%	65 4.0%

Table showing the number and percentage of significant overlapping genes. *γ* refers to the number of genes compared against and is the number of unique genes within all the significant subnetworks of the disease datasets. The gene overlap refers to the percentage gene overlap between the two datasets of a disease for SNet (column 3) and GSEA (column 4).

**Table 4:** Number and percentage of significant overlap genes with t-test

		SNet	t-test	t-test
Leukaemia	Num Genes Genes overlap	*δ* =84 91.30%	1239 73.01%	84 14.29%

ALL subtype	Num Genes Genes overlap	*δ* =75 93.01%	1072 60.20%	75 57.33%

DMD	Num Genes Genes overlap	*δ* =45 69.23%	1319 49.60%	45 20.00%

Lung	Num Genes Genes overlap	*δ* =65 51.18%	2091 65.61	65 26.16%

Table showing the number and percentage of significant overlapping genes. *γ* refers to the number of genes compared against and is the number of unique genes within all the significant subnetworks of the disease datasets. The gene overlap refers to the percentage gene overlap between the two datasets of a disease for SNet (column 3) and t-test (column 4: for genes at P *leg* 0.05; and column 5: for top *γ* significant genes).

**Table 5:** Number and percentage of significant overlap genes with SAM

		SNet	SAM	SAM
Leukaemia	Num Genes Genes overlap	*δ* =84 91.30%	1305 49.96%	84 22.62%

ALL subtype	Num Genes Genes overlap	*δ* =75 93.01%	464 81.25%	75 49.33%

DMD	Num Genes Genes overlap	*δ* =45 69.23%	126 76.98%	45 42.22%

Lung	Num Genes Genes overlap	*δ* =65 51.18%	966 65.61	65 24.62%

Table showing the number and percentage of significant overlapping genes. *γ* refers to the number of genes compared against and is the number of unique genes within all the significant subnetworks of the disease datasets. The gene overlap refers to the percentage gene overlap between the two datasets of a disease for SNet (column 3) and SAM (column 4: for genes at P *leq* 0.05; and column 5: for top *γ* significant genes).

### Size of subnetworks

This section shows that the size of the subnetworks obtained by our algorithm is significantly larger than those obtained from the t-test algorithm. We first obtain a ranked gene list for each dataset using the t-test algorithm. Assuming once again that the total number of genes present within the significant subnetworks for a dataset *i* is *δ_i_*, we extract the top *δ_i_* genes from the ranked gene list for each dataset *i*. Lastly, we calculate the size of the subnetworks formed by these top *δ_i_* genes. We compare these sizes with subnetworks formed by SNet. The results in Table 6 show that the subnetworks obtained by SNet are large (which always contain at least 5 genes and many contain more than 8 genes), while subnetworks obtained by the t-test are small in size (which generally contain 2 or 3 genes and are always no more than 5 genes).

**Table 6:** Size of largest subnetworks from t-test

Disease	*γ*	Num genes (t-test)				Num genes (SNet)			
		2	3	4	5	5	6	7	≥ 8
Leukaemia	84	8	1	0	0	2	3	2	1
Subtype	75	5	1	1	1	1	0	1	6
DMD	45	3	1	0	0	1	0	0	5
Lung	65	3	2	1	0	5	3	0	1

Table comparing the size of the subnetworks obtained from the t-test and from SNet. The first column shows the disease that is being considered and the second column shows the number of genes used to create the subnetworks. The third column (which comprises additionally of 4 subcolumns) depicts the number of genes present within each subnetwork for the t-test. Similarly the fourth column depicts the number of genes present within each subnetwork for SNet. So for instance in the leukaemia dataset, we have 8 subnetworks with size 2 genes, 1 subnetwork with size 3 genes for the t-test. For SNet, we have 2 subnetworks with size 5 genes, 3 subnetworks with size 6 genes, 2 subnetworks with size 7 genes and 1 subnetwork with a size of ≥ 8 genes

### Validity of genes within each subnetwork

To check the validity of the subnetworks selected, we compare the genes present within each subnetwork with those deemed significant by the t-test. A high percentage would mean that the genes within our captured subnetworks are highly consistent to established methods such as t-test, yet at the same time rejecting genes that are non-consistent over datasets (hence likely to be false positives). Table 7, 8, 9, Table 10 show the different subnetworks found significant within their respective disease sets. The corresponding percentage depicts the percentage of genes present within the subnetwork which are also significant by the t-test (taken with a p-value threshold of 0.05). We can observe from the tables that the bulk of the subnetworks have a high consistency percentage, falling between 70% to 100%.

**Table 7:** Percentage of genes from subnetworks for the leukaemia dataset which are also considered significant by t-test

Subnetwork name	Percentage
leukaemia_B Cell_VAV1	81.82%
leukaemia_Purine metabolism_NP	83.33%
leukaemia_Phosphatidylinositol signaling_PLCG2	100.00%
leukaemia_Regulation of actin cytoskeleton_RAC1	57.14%
leukaemia_Proteasome Degradation_UBC	100.00%
leukaemia_Regulation of Actin Cytoskeleton_RAC1	57.14%
leukaemia_B Cell_NFKB1	80.00%
leukaemia_Regulation of actin cytoskeleton_CSK	75.00%
leukaemia_B Cell Receptor Signaling_POU2F2	75.00%
leukaemia_IL6 Signaling_IL8	75.00%
leukaemia_Focal Adhesion_ACTB	100.00%

Table depicting the percentage of genes from subnetworks which are also significant for the t-test. The first column depicts the name of the subnetwork considered. The second column depicts the percentage of genes from that subnetwork which are also deemed significant for the t-test. (leukaemia datasets [26,27])

**Table 8:** Percentage of genes from subnetworks for the ALL subtype which are also considered significant by t-test

Subnetwork name	Percentage
MLLBCR_Fatty acid metabolism_ACAA1	28.57%
MLLBCR_Valine, leucine and isoleucine degradation_HSD17B10	40.00%
MLLBCR_B Cell_BLNK	72.73%
MLLBCR_Valine, leucine and isoleucine degradation_HSD17B10	33.33%
MLLBCR_B cell receptor signaling pathway_BLNK	72.73%
MLLBCR_Acute myeloid leukaemia_FLT3	44.44%
BCR_Chronic myeloid leukaemia_ABL1	75.00%
BCR_Fc Epsilon RI Signaling_PIK3C2B	70.00%
BCR_T Cell Receptor Signaling Pathway_RASA1	44.44%

Table depicting the percentage of genes from subnetworks which are also significant for the t-test. The first column depicts the name of the subnetwork considered. The second column depicts the percentage of genes from that subnetwork which are also deemed significant for the t-test. (ALL Subtype datasets [28,29])

**Table 9:** Percentage of genes from subnetworks for the DMD dataset which are also considered significant by t-test

Subnetwork name	Percentage
DMD_Tight junction_RHOA	87.50%
DMD_Integrin Signaling_TTN	75.00%
DMD_ECM-receptor interaction_SDC3	88.89%
DMD_Tight junction_RHOA	85.71%
DMD_Leukocyte transendothelial migration_ACTB	83.33%
DMD_Actin Cytoskeleton Signaling_MYL9	78.57%
DMD_Calcium signaling pathway_CALM1	80.00%

Table depicting the percentage of genes from subnetworks which are also significant for the t-test. The first column depicts the name of the subnetwork considered. The second column depicts the percentage of genes from that subnetwork which are also deemed significant for the t-test. (DMD datasets [16,17])

**Table 10:** Percentage of genes from subnetworks for the lung dataset which are also considered significant for the t-test

Subnetwork name	Percentage
SNet_Notch signaling pathway_NOTCH3	100.00%
SNet_ECM-receptor interaction_SDC1	69.23%
SNet_Adherens junction_CTNNB1	100.00%
SNet_Tyrosine metabolism_ADH1B	100.00%
SNet_Phenylalanine metabolism_ALDH3B1	100.00%
SNet_Tryptophan metabolism_WBSCR22	80.00%
SNet_Natural killer cell mediated cytotoxicity_TNFSF10	60.00%
SNet_Insulin Recpetor Signaling_AKT3	100.00%
SNet_Glycogen Metabolism_PYGM	60.00%

Table depicting the percentage of genes from subnetworks which are also significant for the t-test. The first column depicts the name of the subnetwork considered. The second column depicts the percentage of genes from that subnetwork which are also deemed significant for the t-test. (Lung datasets [14,15])

### Biological relevance of subnetworks

**Figure 2:**
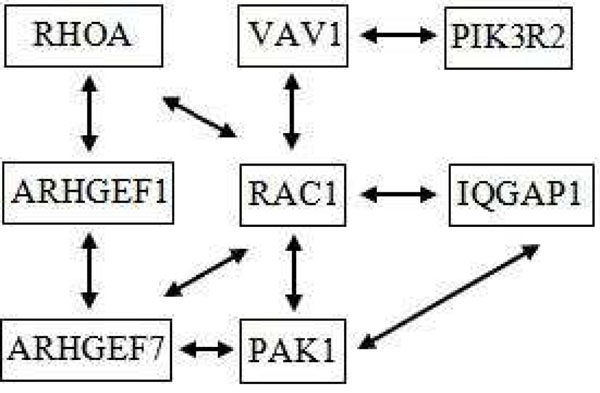
**Sample subnetwork from leukaemia dataset.** A sample subnetwork from leukaemia dataset [26,27].

Two small sample subnetworks are chosen here to show the biological significance of the results obtained. The first which we describe below (and in Figure 2) is generated from the leukaemia dataset. The genes within this subnetwork are very substantially supported by literature with respect to their role in leukaemia. For instance, the gene RAC (which regulates a diverse array of cellular events) is referenced in [30,31] as having an effect on leukaemia. Other genes within the network are Rhoa (regulates the actin cytoskeleton in formation of stress fibers) in [32,33], Vav1 (plays a major role in development and activation of T-cell and B-cell blood cells) in [34] and IQGAP (regulates cell adhesion, morphology and motility) in [35].

**Figure 3:**
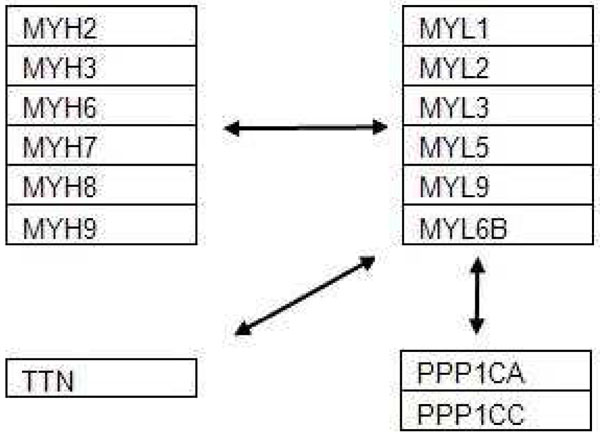
**Sample subnetwork from leukaemia database.** A sample subnetwork from DMD dataset [16,17].

The next subnetwork shown in Figure 3 is generated from the DMD disease datasets, and is taken from the Apoptosis pathway. Results from our algorithm indicated that the genes groups MYL and MYH are significantly differentiately expressed between the DMD patients and the normal patients. MYH (myosin, heavy chain) and MYL (myosin, light chain) are known to be major gene groups involved in release of mechanical energy allowing muscles to contract. These genes are heavily quoted in literature with regard to their involvement in the disease DMD: MYH3 and MYH8 [17], MYH6 [36], MYH7 [37], MYL1, MYL2, MYL3, MYL4, MYL5, MYL6 and MYL9 [36]. In addition, the gene titin was identified. Titin is a gene which encodes a large protein of the spinal skeletal muscles and its mutation is widely found to occur in various types of muscular dystropy [38-41].

## Conclusions

Microarray experiments are crucial because they measure the behaviour of individual genes with respect to diseases or treatments. Results from these experiments are heavily scrutinised to obtain biological insights into the occurrence of diseases or the effectiveness of certain types of treatments. In order to provide more indepth analysis to experiments, contemporary algorithms have incorporated biological information into their analysis so that the analysis can be more descriptive and hopefully useful to the researchers. Our techniques have taken this approach one step further. Firstly, we no longer consider prior biological knowledge as a separate aspect of microarray analysis. Rather, we take into account the integrity of the biological information that is being provided into the algorithm for analysis. Secondly, our algorithm uses both the gene-gene interaction information and pathway information in our analysis. Because of these two enhancements, we are able to generate subnetworks in real-time according to the responses of the microarray experiments. These contributions help us avoid some of the potential caveats present within microarray experiments.

We are certainly not the first to integrate gene-expression data with gene-gene relationships. GNEA [42] is one such example. GNEA uses a global protein-protein interaction network, finds subnetworks that correspond to regions of significantly differentially expressed genes; these subnetworks are called HSNs in the paper. GNEA then determines which gene sets in a library of gene sets are significantly enriched in HSNs. There are two possible shortcomings in this approach. Firstly, in using a single global protein interaction network, GNEA makes the biological assumption that the local behaviour of proteins can be translated in a similar fashion globally and that gene expression levels are in a tight correspondence to protein levels (which is not generally true). A similar issue is raised in [7] where the authors argued that proteins which are very well connected have an extremely high chance of obtaining a low p-value and being ranked as significant. Because of the high connectivity of such proteins, they are liable to be involved in various disjoint biological processes, leading to the error of combining independent subnetworks through these proteins. To prevent such scenarios, we instead implemented our algorithm via identifying localised gene-gene subnetworks within pathways. Secondly, while a gene set that is significantly enriched in HSNs is likely to be relevant, a large gene set may not be found significantly enriched in HSNs even though it may have contained a subset that is significantly enriched. This is also an issue that we find in GSEA.

We obtain a low result overlap from GSEA possibly because the pathways from PathwayApi are very large and GSEA relies on a large portion of a pathway to exhibit a correlated change. Hence when only a subset of a pathway demonstrates differential expression, GSEA may be unable to pick this up. We verified this hypothesis by feeding into GSEA subnetworks that we found from our algorithm into the leukaemia datasets. Indeed GSEA was then able to obtain significant subnetworks that overlapped.

In addition, we show that our technique generates significant subnetworks and genes that are more consistent across datasets compared to the other popular methods available (GSEA, t-test and SAM). The large size of subnetworks which we generate indicates that they are generally more biologically significant (less likely to be spurious). To validate our results, we show that most of our genes from the generated subnetworks have also been considered significant by the t-test. In addition, we have chosen two sample subnetworks and validated them with references from biological literature. This shows that our algorithm is capable of generating descriptive biologically conclusions.

Our final contribution lies in our ability to create connected components (of known pathways) in real time based on microarray data. This allows us to obtain connected components according to the microarray data. Both GNEA and GSEA use fixed gene sets and determines if these gene sets are significant or not. These techniques assume that a gene set is significant only if a substantial proportion of the genes within the gene set is significant. This assumption might not be valid because there are instances where only part of a gene set becomes significant; and it would probably go unnoticed if most of the rest of the genes are unaffected. Our ability to create connected components based on the microarray data of the phenotypes—and use these as gene sets—ensures that we have sufficient granularity to capture portions of pathways or gene sets that are affected.

## Methods

Overview Let the phenotype of interest be *d* and the remaining phenotypes be labelled as ¬*d*. We first extract genes which are highly expressed within this phenotype *d* from the microarray experiment. This set of genes is next segregated into their respective subnetworks using apriori biological information from the pathway repository [25]. This gives us a list of subnetworks *cc* (whose genes are highly expressed) within *d*. A score (depending on the size of the subnetwork and its consistency among the patients) is next calculated and assigned to each subnetwork. Finally we estimate the p-value of every single subnetwork within the list and keep those which are significant. This is elaborated in the following steps:

**Step 1: Subnetwork extraction** We create a ranked gene list for each patient within a phenotype according to the gene expression level of that patient. From this ranked gene list we extract only the top *α*% of genes for each patient. This condensed gene list is referred to as *G_P_i__* for the *i^th^* patient *P_i_*. We next iterate across gene lists *G_P_i__* only for patients of phenotype *d*, extracting only genes which appear in more than *β*% of the patients of phenotype *d*. This creates a list of genes *GL* which turns up highly expressed across most of the patients of phenotype *d*. Finally, using the programmatic interface of PathwayAPI, gene list *GL* is segregated into the respective subnetworks. In our experiments, *α* is taken to be 10 and *β* to be 50.

**Figure 4:**
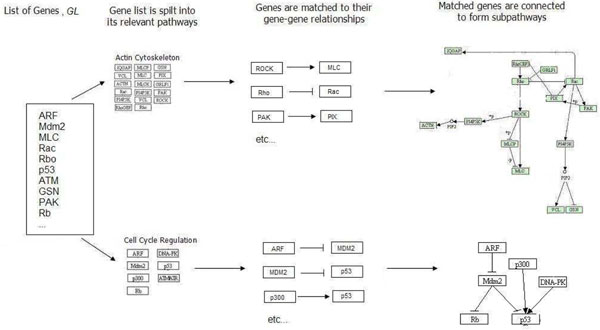
**Sample subnetwork formation.** An example of how we form subnetworks from a sample pathway with its genes.

To segregate *GL* into the different subnetworks, we first split gene list *GL* into its pathways and the gene-gene relationships within these pathways. (We highlight that a gene is allowed to appear in more than one pathway.) Next, by treating each gene as a vertex and each gene-gene relationship as an edge, we can easily locate the connected components (subnetworks) formed by these edges (gene-gene relationships) and vertices (genes) in each pathway. This process is illustrated in Figure 4.

**Step 2: Subnetwork scoring** For each subnetwork *sp* within *cc* and for each patient *P_i_* (regardless of phenotype), we compute the overall expression level of *sp* in *P_i_* by(1)

Here, *g* denotes a gene in the subnetwork *sp* that is highly expressed (top *α%*) in patient *P_i_* ; *k* is the number of patients of phenotype *d* who have gene *g* highly expressed (top *α%*); and *n* is the total number of patients of phenotype *d*.

Let *P*_1_, …, *P_n_* be patients of phenotype *d;* and *P_n_*_+1_, …, *P_m_* be patients of other phenotypes ¬*d*. We assign two score vectors *Ssp_sp_*_,_*_d_* and *Ssp*_*sp*,¬*d*_ respectively for these two groups of patients, where(2)

The t-statistics is now calculated between these two vectors, creating a final score for each subnetwork *sp* within *cc*. We call this score *Ssp_sp_*_,_*_t_*.

**Figure 5:**
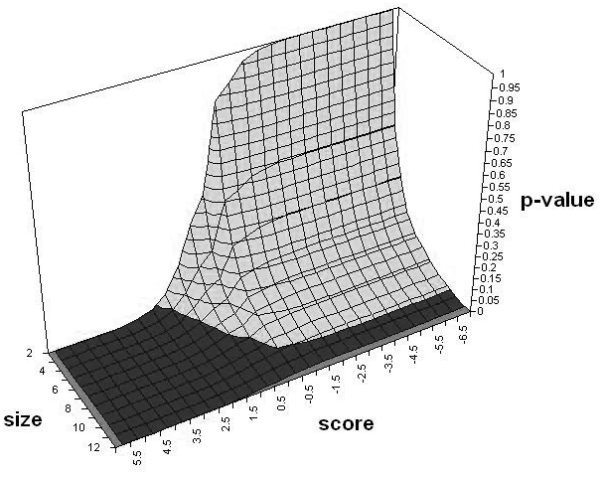
Sample null distribution of subnetworks according to the size and score.

**Step 3: Subnetwork significance** We repeat Steps 1 and 2 for all the phenotypes in the dataset to extract a list of subnetworks SN. The significance of the observed subnetworks is estimated by randomly permuting the phenotypes labels, re-extracting the subnetworks and recomputing their t-statistics scores. This generates a null distribution for the score and size of the subnetworks. The p-value of each subnetwork is then calculated relative to this null distribution. The null hypothesis being that for a subnetwork obtained of size *|sp|* and score *Ssp*_*sp*,*t*_, the subnetwork is not significant. An example of such a distribution is seen in Figure 5. In detail, the procedure is as follows:

A Randomly swap the phenotype labels of the patients, recreating the subnetworks and recalculating their t-statistics scores.

B Repeat [A] for 1,000 permutations. This creates a two dimensional histogram of the scores and sizes of the subnetworks.

C Estimate the nominal p-value of each subnetwork by using the histogram created in point [B].

Finally, we consider subnetworks whose p-value was sufficiently small (≤ 0.05) to be significant. Doing so would provide us with an independent set of significant subnetworks *SN* for each dataset. Using our algorithm, we have managed to show that we are able to obtain consistent significant subnetworks across different datasets of the same disease.

## Competing interests

The authors declare that they have no competing interests.

## Authors' contributions

DS developed the software. DS and LW wrote the manuscript. All authors contributed to the design of analytical algorithms. All authors read and approved the final manuscript.
